# Increased perceived self-efficacy facilitates the extinction of fear in healthy participants

**DOI:** 10.3389/fnbeh.2015.00270

**Published:** 2015-10-16

**Authors:** Armin Zlomuzica, Friederike Preusser, Silvia Schneider, Jürgen Margraf

**Affiliations:** Mental Health Research and Treatment Center, Ruhr-University BochumBochum, Germany

**Keywords:** self-efficacy, extinction, fear conditioning, exposure therapy, anxiety disorders, self-regulation, top-down control

## Abstract

Self-efficacy has been proposed as an important element of a successful cognitive behavioral treatment (CBT). Positive changes in perceived self-efficacy have been linked to an improved adaptive emotional and behavioral responding in the context of anxiety-provoking situations. Furthermore, a positive influence of increased self-efficacy on cognitive functions has been confirmed. The present study examined the effect of verbal persuasion on perceived self-efficacy and fear extinction. Healthy participants were subjected to a standardized differential fear conditioning paradigm. After fear acquisition, half of the participants received a verbal persuasion aimed at increasing perceived self-efficacy. The extinction of fear was assessed immediately thereafter on both the implicit and explicit level. Our results suggest that an increased perceived self-efficacy was associated with enhanced extinction, evidenced on the psychophysiological level and accompanied by more pronounced decrements in conditioned negative valence. Changes in extinction were not due to a decrease in overall emotional reactivity to conditioned stimuli (CS). In addition, debriefing participants about the false positive feedback did not affect the processing of already extinguished conditioned responses during a subsequent continued extinction phase. Our results suggest that positive changes in perceived self-efficacy can be beneficial for emotional learning. Findings are discussed with respect to strategies aimed at increasing extinction learning in the course of exposure-based treatments.

## Introduction

The concept of self-efficacy refers to the individual's perceived belief to cope effectively with upcoming situations and problems (Bandura, 1997). A higher level of self-efficacy can increase the individual's belief that his/her behavior will more likely produce a positive outcome within a given situation (Bandura, 1997; Maddux, 1999). According to Bandura (1997) four different sources of self-efficacy information can be differentiated (i.e., as a result of mastery experience, vicarious experience, persuasion, and physiological and affective states).

The beneficial impact of increased perceived self-efficacy on behavior has been confirmed across different domains of research. For instance, a positive relationship between the level of self-efficacy and sports performance (Moritz et al., 2000), the likelihood to engage in healthy behavior (Schwarzer and Fuchs, 1995), but also the ability to cope adaptively with stressful experiences (McFarlane et al., 1995) has been demonstrated. Self-efficacy has also been linked to cognitive performance as demonstrated in different verbal, mathematical, and spatial tasks (Lent et al., 1997; Paunonen and Hong, 2010).

The concept of self-efficacy has received a great deal of attention in clinical research. Perceived self-efficacy or the confidence in being able to refrain from smoking predicts smoking cessation outcome and has been considered a potential mechanism underlying effective smoking abstinence (Gwaltney et al., 2005; Marlatt and Donovan, 2005; Schnoll et al., 2011). A decreased self-efficacy has been discussed as a cognitive precursor or a component of anxiety, phobia, and depression (Comunian, 1989; Williams, 1995) and is associated with a greater severity of anxiety (Richards et al., 2002; Thomasson and Psouni, 2010) and an increased tendency to use dysfunctional coping strategies when confronted with anxiety-provoking situations (Thomasson and Psouni, 2010).

A positive change in perceived self-efficacy in the course of a cognitive behavioral treatment (CBT) might constitute a critical component of a successful therapeutic outcome. Increases in self-efficacy go along with reductions in anxiety symptoms following treatment (Bouchard et al., 2007; Gaudiano and Herbert, 2007; Delsignore et al., 2008). Likewise, the amount of cognitive reappraisal self-efficacy, defined as the belief that one can effectively apply emotion regulation strategies during exposure to anxiety-provoking stimuli and situations, significantly determines anxiety symptoms reductions (both immediate and long-term) following CBT (Goldin et al., 2012). Findings from these and other studies implicate that increments in self-efficacy beliefs may constitute an important mechanism through which CBT exerts its beneficial effects on fear and avoidance (Bouchard et al., 2007; Goldin et al., 2012; Gallagher et al., 2013).

The exact role of self-efficacy in CBT remains elusive. CBT for anxiety disorders usually involves a combination of exposure and a set of cognitive strategies in order to modify the patient's negative expectations and interpretations in the context of anxiety-provoking situations. Based on the research so far, it is difficult to ascertain whether increases in self-efficacy arise from symptom relief experienced by patients during CBT or whether different levels of self-efficacy in patients determine their range of emotional and/or behavioral responding in the context of anxiety-provoking situations. Most studies so far have utilized correlational designs to examine the relationship between self-efficacy and CBT outcome (Bouchard et al., 2007; Gallagher et al., 2013). Clearly, more research with experimental designs is needed to describe the exact link between increases in self-efficacy and CBT outcome.

Exposure, a core component of CBT, can lead to an enduring symptom relief in anxiety disorders (Ruhmland and Margraf, 2001a,b,c; Vögele et al., 2010). During exposure, patients are given the opportunity to reevaluate the significance of a stimulus while extinction learning has been proposed to mediate this form of so called “corrective learning” (Craske et al., 2008, 2014; Vervliet et al., 2013). Given that low self-efficacy has been linked to an increased tendency of anxious individuals to use dysfunctional coping strategies in anxiety-related situations (Thomasson and Psouni, 2010) it is reasonable to assume that changes in self-efficacy might affect extinction learning as shown previously for other forms of learning (McDougall and Kang, 2003). This might be of special importance for understanding how self-efficacy beliefs in its interaction with deficient fear extinction and elevated fear acquisition (Briscione et al., 2014; Mosig et al., 2014) contribute to the development of anxiety and stressor-related disorders. Likewise, it offers the possibility to examine how specific interventions aimed at enhancing self-efficacy can be applied to exposure to yield more enduring and stable therapy benefits (Rothbaum and Davis, 2003; Craske et al., 2008, 2014; Norrholm and Jovanovic, 2010; Vervliet et al., 2013).

In this instance, it was recently shown that self-efficacy beliefs can indeed be systematically manipulated via persuasive verbal feedback and that this manipulation affects the memory for both aversive (Brown et al., 2012b) as well as personally relevant (Brown et al., 2012a) events. In particular, following a high or low self-efficacy induction, participants with a high-self efficacy belief recalled fewer negative intrusions and showed a reduction in attentional bias associated with remembering aversive stimuli (Brown et al., 2012b). In a similar vein, it was demonstrated that students who were led to believe they possessed high self-efficacy showed an increase in episodic memory performance and problem solving capacity (Brown et al., 2012a). These findings indicate that self-efficacy beliefs can be mediated via positive and/or negative persuasive feedback beliefs (Bandura, 1997), which might (in)directly influence learning and/or retrieval of emotionally relevant information. On the neurobiological level, such effects might be comparable to those elicited via top-down modulation of fear learning and extinction by means of intentional cognitive regulation strategies. Reappraisal, for instance, has been shown to alter fear extinction via activation of downstream pathways, i.e., frontal regions including the ventromedial prefrontal cortex, which are implicated in the inhibition of fear responses (Buhle et al., 2014; Schiller and Delgado, 2010).

The present study sought to determine whether (similar to other top-down regulation strategies) the manipulation of self-efficacy beliefs via positive verbal persuasion might also affect fear extinction learning. Hence, the major aim of this study was to extend previous findings and examine the effect of an experimentally-induced increased self-efficacy on the extinction of learned fear. Since fear extinction is becoming widely accepted as a translational tool for exposure-based treatments, our findings might provide more insights into the relation between self-efficacy levels and CBT outcome. Moreover, by investigating the effect of increased self-efficacy on subsequent fear extinction and the neuronal circuitry involved we might determine whether an increase in self-efficacy can be used to enhance exposure-based treatments (Craske et al., 2008, 2014; Vervliet et al., 2013).

## Materials and methods

### Participants

The sample was recruited via postings in social media networks or announcements on bulletin boards at the campus of the Ruhr-University Bochum. Participants reporting current or previous mental diseases, psychological or pharmacological treatment for mental diseases, as well as severe acute or chronic somatic diseases were not eligible for participation. A total of 57 subjects (28 males, 29 females) participated in this study. Data from nine participants were excluded because they failed to acquire conditioning (e.g., higher CS-UCS contingency and CS valence ratings for the unreinforced as compared the reinforced conditioned stimuli (CS) after fear acquisition) or reported that they did not believe the manipulated self-efficacy feedback. Hence, our final analysis comprised data from 48 participants, who were randomly assigned to either the experimental group (*n* = 24, 50% females) or the control group (*n* = 24, 62.5% females). Demographic characteristics of the groups are displayed in Table 1. Subjects received either 15€ or 1.5 course credits as compensation for time and travel. All experimental procedures were approved by the local ethics committee (Ethical committee of the Ruhr-University of Bochum, Germany) and carried out in accordance with the Declaration of Helsinki. All participants provided written informed consent.

**Table 1:** **Demographic characteristics of the experimental and control group**.

**Variable**	**EG**	**CG**
	***M (SD)***	***M (SD)***
Age	23.63 (5.22)	24.00 (5.48)
% Female	50	62.5
Use of contraceptives (females only) %	75	66.7
DASS (stress)	6.83 (4.23)	6.67 (4.64)
DASS (anxiety)	1.96 (2.31)	2.42 (2.78)
DASS (depression)	2.67 (3.38)	3.38 (3.83)
DASS (total)	11.46 (8.52)	12.46 (10.30)
UCS valence	80.33 (13.93)	81.38 (12.93)
UCS intensity (mA)	5.79 (4.10)	4.84 (3.82)

*EG: positive feedback (self-efficacy induction); CG: no feedback (no self-efficacy induction). Based on N = 48 subjects (n_EG_ = 24, n_CG_ = 24)*.

### Experimental design

#### Fear conditioning

Each participant underwent a differential fear conditioning procedure according to a modified procedure previously described by Blechert et al. (2007) and Michael et al. (2007). Two inkblot pictures, either black and white or yellow and red in color, served as the reinforced CS+ and unreinforced CS− in a counterbalanced manner. A 500 ms mild electrical stimulation delivered to the skin of the lower arm constituted the UCS. The entire fear conditioning procedure consisted of a habituation, acquisition, extinction, and a continued extinction phase. A break of 15 min was imposed after acquisition as well as after extinction to administer the first and second experimental manipulation (see Section Experimental Manipulations). Three trials of CS+ and CS− were presented during habituation. In the acquisition phase, the CS+ and CS− were again presented 10 times each while the CS+ co-terminated with the UCS on a 60% reinforcement schedule (to extend the time course of fear responses during the subsequent extinction phase, see Haselgrove et al., 2004). The extinction phase consisted of 20 trials (10 CS+ and 10 CS−) without any UCS. After the second experimental manipulation, extinction was continued and another set of 6 (3 CS+ and 3 CS−) trials, respectively, was presented. During all phases, the CS+ and CS− were displayed for 8 s each and presented in pseudorandom order. The duration of the randomly generated inter-trial interval was between 16 and 20 s. Performance measures included skin conductance responses (SCRs) as well as CS valence and CS−UCS contingency ratings.

#### Experimental manipulations

##### Experimental manipulation 1: the effect of verbal persuasion on fear extinction

**Figure 1:**
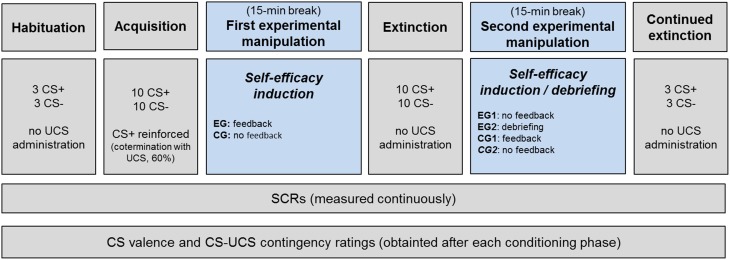
**Experimental design**. The fear conditioning procedure consisted of a habituation, acquisition, extinction, and continued extinction phase, with 15-min breaks imposed after acquisition as well as extinction. After fear acquisition (first experimental manipulation), the experimental group (EG) received a verbal feedback to induce self-efficacy expectations, whereas the control group (CG) received none. After extinction (second experimental manipulation), the false feedback was revised for half of the experimental group (EG2), whereas it was administered to half of the control group (CG1). Dependent measures included valence and contingency ratings as well as skin conductance responses (SCRs).

The present study employed two experimental manipulations. The experimental design is illustrated in Figure 1. The first experimental manipulation was used to examine whether it is possible to alter fear extinction by adding verbal persuasion (vs. no verbal persuasion) aimed at increasing self-efficacy beliefs. For this purpose, all participants first underwent a fear acquisition phase. Subsequently, half of the participants (i.e., the experimental group, EG) received a slightly modified version of the positive verbal feedback used by Brown et al. (2012b). Specifically, they were told that, based on the way in which they responded to the questionnaires and their physiological responses during the task, they had been identified as being in the top 1% of “copers” and to possess excellent abilities when dealing with stressful situations (for more details, see Brown et al., 2012b). By contrast, a verbal feedback was not administered to the control group (CG).

##### Experimental manipulation 2: the effect of debriefing and verbal persuasion on continued extinction

The purpose of the second experimental manipulation was to investigate whether a change in continued extinction (or a return in conditioned responses) would occur in the EG after debriefing participants about the false positive verbal feedback. Thus, after the extinction phase, half of the experimental group (i.e., EG2) were debriefed about the nature of the false feedback they had received earlier, whereas the remaining subjects (i.e., EG1) did not receive any feedback.

Finally, we investigated whether the application of the verbal persuasion (i.e., the identical false positive verbal feedback as described above) is effective in promoting extinction when applied after the extinction had already been initiated. Here, participants from CG were randomly assigned either to the “false feedback” condition (i.e., CG1, receiving the identical instruction as EG, see descriptions above) or “no feedback” condition (CG2). Hence, the CG2 subgroup received no feedback at any experimental stage (but see Experimental Design).

Importantly, the delays imposed between the respective conditioning phases (i.e., acquisition and extinction; extinction and continued extinction) were identical among groups regardless of whether a verbal feedback was delivered.

### Apparatus

The experiment took place in a sound-attenuated room adjacent to a control room where the experimental apparatus was stationed. Stimuli as well as rating scales of the conditioning procedure were presented on a 19-inch computer screen (Computer GmbH&Co KG, Marl, Germany) using Presentation software, version 16.1 (Neurobehavioral Systems, Inc., USA). Ag/AgCl electrodes placed on the lower left arm delivered the electrical stimulation generated by a Constant Current Isolated Stimulator PS3 (Digitimer Ltd., Welwyn Garden City, England). SCRs were measured with 5 mm inner-diameter Ag/AgCl electrodes filled with non-hydrating electrodermal response paste and positioned at the index and middle finger of the non-dominant hand. Signals were recorded and digitzed at a sampling rate of 1000 Hz in a continuous mode utilizing a 16-Bit BrainAmp ExG Amplifier and Brain Vision Recorder software, version 1.2 (Brain Products GmBH, Gilching, Germany).

### Assessments

#### Questionnaires

Prior to the conditioning procedure, selected items from the self-report *Depression Anxiety Stress Scales* (DASS; Lovibond and Lovibond, 1995) were applied to measure acute symptoms of anxiety, depression and stress on a 4-point Likert scale (0 = did not apply to me at all, 3 = applied to me very much, or most of the time). Furthermore, participants' perceived ability to cope with emotions, solve problems and gain social support was assessed using the *Resilience Appraisal Scale* (RAS; Johnson et al., 2010), which was filled in after the first false feedback. The RAS consists of 12 items scored on a five-point Likert scale (1 = strongly disagree, 5 = strongly agree). Before as well as after each false feedback, subjects indicated their current level of distraction and excitement, their mood (positive and negative), as well as their perceived self-efficacy on five *Visual Analogue Scales* (VAS), with each scale ranging from 1 (minimal) to 10 (maximum).

#### CS-valence and CS-UCS contingency ratings

After each phase of the conditioning procedure, ratings of CS valence (how pleasant/unpleasant do you feel when you see this picture?) and CS-UCS contingency (do you think that this picture is paired with an electrical stimulation?) were obtained using VAS presented on the screen. Subjects had to mark their rating with the cursor of the mouse. The anchor labels for the valence ratings and contingency ratings ranged from 0 (very pleasant) to 100 (very unpleasant) and 0 (extremely unlikely) to 100 (extremely likely), respectively.

#### Skin conductance responses

SCRs for each trial were calculated by subtracting the mean skin conductance level (SCL) during the 1000 ms prior to CS onset (baseline) from the maximum SCL recorded during the 8 s after CS onset. SCRs were z-transformed to attain a normal distribution.

### General procedure

Each participant was welcomed by the experimenter, who was dressed in a laboratory coat to increase his credibility, and led into the experimental room. Participants were seated upright in a comfortable chair in front of a computer screen and informed about the content of the experiment. Specifically, they were told that the experiment would involve the presentation of two different pictures and that one of these pictures may be paired with an electrical stimulation. In addition, to increase plausibility for the (to-be-implemented) experimental manipulations, participants were told that their physiological responses during the task as well as their responses to the questionnaires would be analyzed continuously by the experimenter in the laboratory room. Subsequently, electrodes for the electrical stimulation and measurement of SCRs were attached to the non-dominant arm. The intensity of the electrical stimulation for the conditioning procedure was adjusted to a sensation level participants experienced as “highly unpleasant but not painful” (adapted from Blechert et al., 2007). After participants had practiced and fully understood the rating scales (i.e., CS valence and CS-UCS contingency), the experimenter left the room and all participants completed the differential fear conditioning procedure (for details see Experimental Design). After each positive false feedback, the respective groups (i.e., EG after the first experimental manipulation, and CG1 after the second experimental manipulation) were asked whether they could identify with the feedback and to mention three keywords on how they cope in stressful situations (cf. Brown et al., 2012b). This was implemented as a manipulation check in order to determine whether these groups believed the false feedback (Brown et al., 2012b).

The experimenter only re-entered the room to deliver the feedback/debriefing or to distribute the VAS during the 15-min breaks of the fear conditioning procedure. At the end of the experiment, electrodes were removed and subjects were informed about the false feedback and were fully debriefed.

### Statistical analyses

Statistical analyses were carried out in IBM SPSS Statistics for Windows, version 20.0 (Armonk, NY, USA: IBM Corp.). Manipulation checks, i.e., induction of self-efficacy, were examined using a series of two-way mixed ANOVAs on each VAS with Group as between-subjects factor and Time (pre vs. post induction) as within-subjects factor. For the habituation, acquisition, and extinction phases, mixed ANOVAs with CS-type (CS+ vs. CS−) as within-subjects factor and group (EG vs. CG) as between-subjects factor were employed to analyze the effects of experimental manipulation 1 on subjective CS valence, CS-UCS contingency ratings and mean SCRs scores. In addition, the within subjects-factor “block” (early vs. late; averaged across the first and last five trials of the CSs, respectively) was added for the extinction and acquisition phases.

With respect to the effects of the second experimental manipulation, the end of extinction (for SCRs only the last extinction block was considered) was compared to continued extinction, using mixed ANOVAs with CS-type and Phase. Analyses were conducted separately for the experimental group (i.e., EG1 and EG2, effects of debriefing) and control group (i.e., CG1 and CG2, effects of late feedback). The critical alpha level was set to 0.05. *Post-hoc* analyses were performed using simple effects analysis and/or Bonferroni-corrected pairwise comparisons.

## Results

Participants who received the self-efficacy induction during the first experimental manipulation (EG) did not differ from control participants (CG) with respect to relevant control variables such as age, gender, stress, or their scores on the DASS and its' subscales, nor with respect to the acquisition phase, all *p* > 0.05 (cf. Table 1).

### Manipulation checks

#### Experimental manipulation 1

Descriptive statistics are displayed in Table 2. With respect to the first experimental manipulation and the self-efficacy scale in particular, significant main effects were found for Time [*F*_(1, 46)_ = 8.639; *p* = 0.005; η${}_{\text{p}}^{2}=0.158$] and Group [*F*_(1, 46)_ = 8.083; *p* = 0.007; η${}_{\text{p}}^{2}=0.149$] as well as their interaction [*F*_(1, 46)_ = 9.312; *p* = 0.004; η${}_{\text{p}}^{2}=0.168$], indicating that the first experimental manipulation was successful. In particular, the experimental group showed an increase in perceived self-efficacy after the experimental manipulation (see Table 2). Moreover, self-efficacy ratings obtained after the experimental manipulation were higher in the experimental group relative to the control group (cf. Table 2). In addition, there was a significant main effect of Group on the positive mood scale [*F*_(1, 64)_ = 4.578; *p* = 0.038; η${}_{\text{p}}^{2}=0.091$] (cf. Table 2).

**Table 2:** **Manipulation checks**.

**Variable**	**First experimental manipulation**		**Second experimental manipulation**			
	**EG**	**CG**	**EG1**	**EG2**	**CG1**	**CG2**
	***M (SD)***	***M (SD)***	***M (SD)***	***M (SD)***	***M (SD)***	***M (SD)***
**PRE INDUCTION**						
Distraction	2.79 (2.48)	2.79 (2.06)	3.83 (2.68)	3.51 (2.24)	3.16 (2.30)	3.89 (1.81)
Excitement	3.91 (2.46)	4.44 (2.09)	1.86 (1.42)	2.95 (2.77)	1.74 (1.57)	2.62 (1.89)
Positive mood	6.94 (2.42)^d, f^	5.68 (1.90)^e, f^	6.88 (2.78)	7.40 (1.88)	6.21 (1.77)^i^	6.57 (1.35)
Negative mood	2.48 (2.98)	2.81 (1.95)	2.51 (3.71)	2.00 (2.56)	1.70 (2.02)	2.37 (1.87)
Self-confidence	6.70 (1.72)^b^	5.86 (1.44)	6.91 (1.73)	7.32 (1.88)	6.28 (1.42)^h^	6.42 (1.52)
**POST INDUCTION**						
Distraction	2.76 (2.37)	3.20 (1.92)	3.49 (2.37)	3.54 (1.69)	2.12 (1.40)	4.24 (2.52)
Excitement	3.79 (2.41)	3.83 (2.07)	2.16 (1.90)	2.07 (2.38)	2.64 (1.68)	2.16 (1.84)
Positive Mood	7.32 (2.28)^d, g^	6.02 (1.77)^e, g^	6.98 (2.83)	7.63 (1.95)	7.00 (1.92)^i^	6.87 (1.46)
Negative Mood	1.94 (2.85)	2.55 (1.99)	2.71 (3.74)	1.28 (1.70)	1.53 (2.12)	2.03 (1.83)
Self-confidence	7.46 (1.57)^b, c^	5.85 (1.51)^c^	6.71 (1.82)	7.46 (1.86)	6.75 (1.55)^h^	6.60 (1.63)
RAS score^a^	51.96 (4.97)	51.54 (5.63)				

*RAS = resilience appraisal scale. First experimental manipulation: early positive feedback (EG) after fear acquisition; Second experimental manipulation: late positive feedback (CG1) and debriefing (EG2) after fear extinction; EG1 (n = 12): early feedback only; EG2 (n = 12): debriefing (early feedback); CG 1 (n = 12): late feedback; CG2 (n = 12): no feedback*.

a *administered only after the first experimental manipulation*.

b−i *pairwise comparisons, significant at p ≤ 0.05*.

#### Experimental manipulation 2

With respect to the effects of the second experimental manipulation (i.e., debriefing and the late positive feedback) on subjects' ratings of perceived self-efficacy, main effects for Group and Time as well as their interaction failed to attain statistical significance (all *p* > 0.05).

Subsequent analyses of simple effects, however, revealed that the group who received the late feedback (CG1) showed higher self-efficacy ratings and a more positive mood after the induction [simple effects of time within CG1, all Pillai's trace ≥ 0.117; *F*_(1, 44)_≥5.836; *p* ≤ 0.02, η${}_{\text{p}}^{2}\geq 0.117$; cf. Table 2]. By contrast, the debriefing (EG2) did not cause a lowered self-efficacy expectation or a less happy mood (all *p*≥0.06).

### Valence ratings

#### Experimental manipulation 1

**Figure 2:**
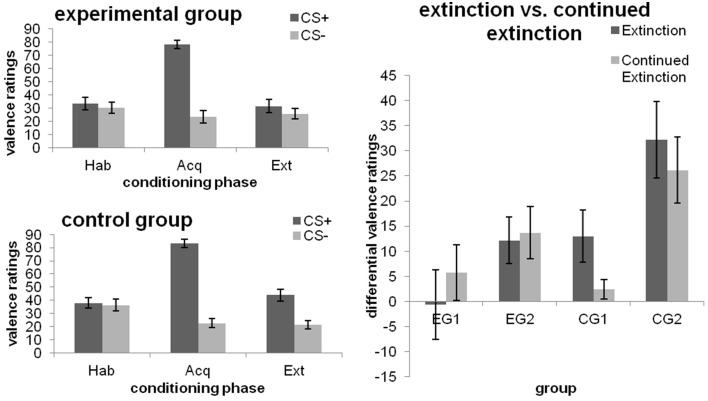
**Valence ratings towards the CSs after the different phases of fear conditioning [habituation (hab), acquisition (acq), extinction (ext); left] as well as changes from extinction to continued extinction [expressed in differential ratings (CS+ minus CS−); right], depicted separately for each group**. Data expressed as means ± 1 SEM; based on *N* = 48 subjects (EG *n* = 24, CG *n* = 24; EG1 *n* = 12, EG2 *n* = 12, CG1 *n* = 12, CG2 *n* = 12).

As expected, no significant differences in valence ratings after habituation were found between groups or the CS+ and CS− (all *p* > 0.05) and the CS+ was rated more negatively than the CS− after fear acquisition [main effect CS-type; *F*_(1, 46)_ = 245.678; *p* < 0.001; η${}_{\text{p}}^{2}=0.842$]. After extinction, a main effect of CS-type was found [*F*_(1, 46)_ = 18.664; *p* < 0.001; η${}_{\text{p}}^{2}=0.289$], which was qualified by a CS-type x Group interaction [*F*_(1, 46)_ = 6.601; *p* = 0.013; η${}_{\text{p}}^{2}=0.125$). Analysis of simple effects revealed that this interaction was not due to significant group differences in the absolute ratings of the CS+ and CS− (both *p*≥0.066), but driven by the fact that the control group, but not the experimental group, continued to rate the CS+ more negatively than the CS− [Pillai's trace = 0.340; *F*_(1, 46)_ = 23.231; *p* < 0.001; η${}_{\text{p}}^{2}=0.340$; cf. Figure 2 (left)].

#### Experimental manipulation 2

##### Effects of debriefing (i.e., EG1 and EG2)

Only the main effect of CS-type [*F*_(1, 22)_ = 5.932; *p* = 0.023; η${}_{\text{p}}^{2}=0.212$] and the interaction between Group and Phase [*F*_(1, 22)_ = 5.592; *p* = 0.027; η${}_{\text{p}}^{2}=0.203$], with a trend towards a significant difference between groups at the end of extinction (*p* = 0.083), were significant. Additional analyses of both groups separately revealed that EG1 did not discriminate among the CSs after both phases (no main effect of CS-type, nor its interaction with Phase; all *p*≥0.130). EG2, however, showed a significant CS+/CS− differentiation [main effect CS-type; *F*_(1, 11)_ = 11.397; *p* = 0.006; η${}_{p}^{2}=0.509$], which did not depend on Phase (CS-type x Phase interaction; *p* = 0.815). In neither group was a main effect of Phase observed (both *p* = 0.114).

##### Effects of the late feedback (i.e., CG1 and CG2)

Main effects of Phase [*F*_(1, 22)_ = 21.259; *p* < 0.001; η${}_{\text{p}}^{2}=0.491$] and CS-type [*F*_(1, 22)_ = 24.879; *p* < 0.001; η${}_{\text{p}}^{2}=0.531$] as well as their interaction [*F*_(1, 22)_ = 5.935; *p* = 0.023; η${}_{\text{p}}^{2}=0.212$] were obtained. The significant CS-type x Group interaction [*F*_(1, 22)_ = 8.490; *p* = 0.008; η${}_{\text{p}}^{2}=0.278$] showed that, averaged across both phases, CG1 and CG2 differed in their reaction towards the CS+ (*p* = 0.025), but not toward the CS− (*p* = 0.52). In addition, CG2, but not CG1, showed significantly higher ratings for the CS+ as compared to the CS− (*p* < 0.001). Interestingly, when groups were analyzed separately, the CS-type × Phase interaction was significant for CG1 [*F*_(1, 11)_ = 5.679; *p* = 0.036; η${}_{\text{p}}^{2}=0.340$], but not for CG2 (*p* = 0.268). Thus, similar to the effects obtained for experimental manipulation 1, CG1 did indeed continue to discriminate among the CSs by the end of extinction (*p* = 0.031), but ceased to do so after continued extinction (*p* = 0.253). By contrast, CG2 rated the CS+ as more aversive than the CS− during both phases (both *p* ≤ 0.002). Results are illustrated in Figure 2 (right).

### CS-UCS contingency ratings

#### Experimental manipulation 1

**Figure 3:**
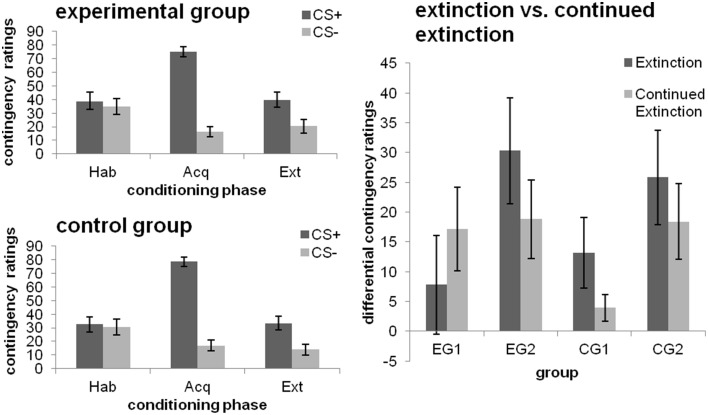
**CS-UCS contigency ratings towards the CSs after the different phases of fear conditioning [habituation (hab), acquisition (acq), extinction (ext); left] as well as changes from extinction to continued extinction [expressed in differential ratings (CS+ minus CS−); right], depicted separately for each group**. Data expressed as means ± 1 SEM; based on *N* = 48 subjects (EG *n* = 24, CG *n* = 24; EG1 *n* = 12, EG2 *n* = 12, CG1 *n* = 12, CG2 *n* = 12).

After habituation, no differentiation between the CS+ and CS− was evident. As depicted in Figure 3 (left), contingency ratings were higher for the CS+ than the CS− after both the acquisition and extinction phase [main effect CS-type; both *F*_(1, 46)_≥22.623; *p* ≤ 0.001; η${}_{\text{p}}^{2}\geq 0.330$]. No effects of Group or a CS-type × group interaction were obtained (all *p* > 0.05).

#### Experimental manipulation 2

##### Effects of debriefing (i.e., EG1 and EG2)

Higher CS-UCS contingency ratings were obtained for the CS+ than the CS− [main effect CS-type; *F*_(1, 22)_ = 18.318; *p* < 0.001; η${}_{\text{p}}^{2}=0.454$]. There was no effect of debriefing, with groups being comparable across both phases (all other main or interaction effects non-significant; *p* = 0. 094).

##### Effects of the late feedback (i.e., CG1 and CG2)

Higher CS-UCS-contingency ratings were obtained for the extinction phase [main effect Phase; *F*_(1, 22)_ = 13.685; *p* = 0.001; η${}_{\text{p}}^{2}=0.383$] and the CS+ [main effect CS-type; *F*_(1, 22)_ = 17.220; *p* < 0.001; η${}_{\text{p}}^{2}=0.439$]. Interactions between CS-type and Phase [*F*_(1, 22)_ = 4.205; *p* = 0.052; η${}_{\text{p}}^{2}=0.160$] as well as CS-type and Group [main effect CS-type; *F*_(1, 22)_ = 3.379; *p* = 0.08; η${}_{\text{p}}^{2}=0.133$] were significant at trend level. Analyses of the simple effect of CS-type for each of the groups separately showed that EG2 discriminated between the CSs after both phases (both *p* ≤ 0.014). By contrast, CG1 did only show a CS+/CS− differentiation after extinction (*p* = 0.048), but not after continued extinction [cf. Figure 3 (right)], which was due to a decrease in UCS-contingency attributed to the CS+ (*p* = 0.006).

### Skin conductance responses

#### Experimental manipulation 1

**Figure 4:**
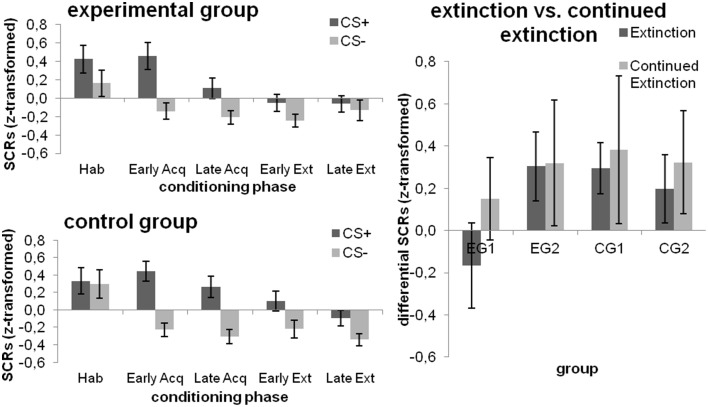
**SCRs towards the CSs during the different phases of fear conditioning [habituation (hab), acquisition (acq), extinction (ext); left] as well as changes from extinction to continued extinction [expressed in differential SCRs (CS+ minus CS−); right], depicted separately for each group**. Data expressed as means ± 1 SEM; based on *N* = 48 subjects (EG *n* = 24, CG *n* = 24, EG1 *n* = 12, EG2 *n* = 12, CG1 *n* = 12, CG2 *n* = 12).

During habituation, subjects did not respond differently towards the CS+ and CS−. There was a significant effect for CS-type [*F*_(1, 46)_ = 42.365; *p* < 0.001; η${}_{\text{p}}^{2}=0.479$] and block [*F*_(1, 46)_ = 6.453; *p* = 0.015; η${}_{\text{p}}^{2}=0.123$] during fear acquisition, with higher SCRs for the CS+ and the early acquisition block. A main effect for CS-type persisted over the extinction phase [*F*_(1, 46)_ = 10.030; *p* = 0.003; η${}_{\text{p}}^{2}=0.179$], yet no other main or interaction effects were significant. However, when the simple main effect of CS-type was tested within each combination of Group and Block, the experimental group did not exhibit any differences in SCRs to the CSs within both the early and late extinction block (both *p* > 0.112), whereas the control group demonstrated higher SCRs toward the CS+ as compared to the CS− in both blocks [both Pillai's trace ≥ 0.085; *F*_(1, 46)_≥4.278; *p* ≤ 0.044; $\eta_{\text{p}}^{2}\geq 0.085$); cf. Figure 4 (left)].

#### Experimental manipulation 2

##### Effects of debriefing (i.e., EG1 and EG2)

All main or interaction effects did not attain statistical significance (all *p*≥0.115).

##### Effects of the late feedback (i.e., CG1 and CG2)

SCRs were higher for the CS+ [main effect CS-type; *F*_(1, 22)_ = 8.978; *p* = 0.007; η${}_{\text{p}}^{2}=0.290$] and for continued extinction [main effect Phase; *F*_(1, 22)_ = 4.317; *p* = 0.05; η${}_{\text{p}}^{2}=0.164$] while none of these effects were subjected to group differences (all *p*≥0.304). Results are displayed in Figure 4 (right).

## Discussion

The main objective of the present study was to examine the impact of an experimental manipulation aimed at increasing self-efficacy beliefs on the subsequent extinction of conditioned fear. We herein provide first evidence that a positive verbal feedback, which increases self-efficacy beliefs, can facilitate fear extinction. Participants who received the experimental induction showed enhanced extinction, as evidenced on the level of psychophysiological responding. Accordingly, they also showed a stronger reduction of conditioned negative valence after extinction relative to participants without the self-efficacy induction. However, the self-efficacy induction had no effect on CS-UCS contingency evaluation.

The results of the present study extend previous findings by Brown et al. (2012a,b) in two ways. First, similar to Brown et al., we could demonstrate that perceived self-efficacy can be experimentally manipulated via verbal persuasion. Second, while Brown et al. showed that such a manipulation can have an impact on autobiographical memory retrieval and problem solving capabilities, our data suggest that increases in perceived self-efficacy can be beneficial for emotional learning. Taken together, these results support the propositions of social-cognitive theories on the role of perceived self-efficacy as an important mediator of cognitive, motivational and affective processes (Bandura, 1997).

The putative mechanisms by which an increased perceived self-efficacy might have affected inhibitory learning performance in our experiment, however, remain elusive. Evidence from previous similar studies (Marquez et al., 2002) suggests that a systematic manipulation via verbal feedback aimed at enhancing perceived self-efficacy can lead to decreased levels of anxiety and arousal. Thus, it is possible that a reduced emotional responding to CSs, during extinction can account for the herein observed effects. Indeed, it has been shown that “state anxiety” changes both the processing of extinguished conditioned responses and the sensitivity with which individuals react to these stimuli during extinction (Vriends et al., 2011). Although the experimental manipulation in our study slightly increased positive mood in our participants, the manipulation had no effect on excitement, or negative mood. It is self-evident that a positive verbal feedback with respect to self-efficacy can lead to concomitant increases in positive mood. However, while Vriends et al. (2011) showed that the induction of a positive emotional state (by means of film induction) leads to a decrease in SCRs to both CSs during extinction, such a response pattern was not evident in our study. In fact, a closer inspection of the SCR data during extinction revealed that the groups did not differ with respect to responding to the CS+ or the CS−. Therefore, it is unlikely that the effects of the positive self-efficacy induction on fear extinction performance (either mediated via increases in self-efficacy, positive mood, or both) are due to a decreased tendency to respond or rather a temporary suppression of emotional reactivity to either the CS+ or CS−. In line with this hypothesis, debriefing participants about the false positive feedback (second experimental manipulation) had no effect on extinguished conditioned responses and thus did not lead to a subsequent revival of extinguished conditioned responses during continued extinction.

A more plausible explanation for the herein observed effect thus might be that an increased perceived self-efficacy altered extinction learning in particular (Craske et al., 2008, 2014; Vervliet et al., 2013). In support of this notion, we found no significant difference in the mean differential SCR to the CS+ and CS− during the late phase of extinction in the group who had received the false positive verbal feedback. Conversely, however, a differential SCR to the CS+ and CS− during the late extinction phase was still existent in individuals without the self-efficacy feedback. Hence, our results indicate that individuals with an increased perceived self-efficacy showed superior extinction learning performance on implicit (i.e., skin conductance responses) as well as subjective measures (i.e., valence ratings) of fear. Interestingly, while individuals with perceived self-efficacy exhibited a more pronounced decrease in conditioned negative valence rating after extinction, no changes with respect to CS-UCS contingency ratings were observed in this group. Hence, self-efficacy enhancement via verbal persuasion might affect the participant's learning about the emotional significance of CSs (reflected on SCR and valence ratings level) while it does not affect the participant's evaluation of stimulus-outcome contingencies. The functional significance of this finding needs further clarification. However, it can be speculated that self-efficacy enhancement engages different control systems (emotional vs. informational) which further rely on distinct neuronal entities to promote fear extinction.

Interestingly, it has been shown that cognitive reappraisal, an emotion regulation strategy used to counteract negative self-beliefs and to increase adaptive emotional reactivity (Goldin et al., 2012), relies on brain systems which are directly involved in fear extinction, including (but not limited to) the dorsolateral prefrontal cortex and dorsal anterior cingulate cortex (Schiller and Delgado, 2010). Moreover, the use of reappraisal techniques during fear conditioning can facilitate fear extinction learning by selectively increasing the inhibitory input of ventromedial prefrontal-amygdala connections (Delgado et al., 2008; Schiller and Delgado, 2010). It would be interesting to investigate whether techniques to increase self-efficacy might constitute another strategy suitable to promote extinction via top-down prefrontal cortex modulation (see also Buhle et al., 2014).

Apart from fear extinction, other studies have already confirmed a positive influence of self-efficacy on learning and memory performance in various other tasks. For instance, self-report measures on perceived self-efficacy have been shown to predict cognitive capabilities (Bouffard-Bouchard, 1990; Paunonen and Hong, 2010) and learning performance rate in procedural tasks (Eyring et al., 1993; Mitchell et al., 1994), as well as academic (Lent et al., 1986) and work-related performances (Stajkovic and Luthans, 1998) in healthy subjects. Moreover, it has been proposed that variations in self-efficacy might account for cognitive differences among young and older adults (Seeman et al., 1996; McDougall and Kang, 2003). Interestingly, older participants show diminished performance in diverse learning and memory tasks which might be at least partly related to age-dependent decreases in perceived learning self-efficacy (Hertzog et al., 1990; Fisk and Warr, 1996; Seeman et al., 1996; McDougall and Kang, 2003). It may be inferred that attempts to increase beliefs about memory efficacy should help older subjects to use mnestic capabilities more effectively in different contexts (McDougall, 1998; Payne et al., 2012). Of course, such a conclusion might be overly simplified as an explanation for the findings of Brown et al. (2012a,b) and our study. While the experimental manipulations aimed to increase perceived self-efficacy were not related to learning self-efficacy (Berry et al., 1989; Berry, 1999) or the specific task domains used, the global positive feedback with respect to self-efficacy might have nevertheless influenced the participant's cognitive resources or the individual's comprehension in the particular paradigm (e.g., McDougall, 1998). For example, Kalpouzos and Eriksson (2013) showed that healthy adults who differ in memory self-efficacy beliefs use different cognitive strategies when encoding episodic memory information. Most importantly, participants with high vs. low self-efficacy beliefs concerning their memory also show a different pattern of brain activation. Hence, it is possible that, similar to “high-memory believers” in the study by Kalpouzos and Eriksson, participants with an increased perceived self-efficacy rely on more efficient mnestic strategies and/or recruit different brain structures during extinction learning. Further research would be needed to test this hypothesis more specifically.

Our findings have important clinical implications. Clinical studies in different anxiety disorders have identified self-efficacy as an important mediator of successful exposure-based treatments (Bouchard et al., 2007; Gaudiano and Herbert, 2007; Delsignore et al., 2008; Gallagher et al., 2013). These studies, however, focused on changes in self-efficacy derived from mastery experiences and provided only correlational evidence on the link between self-efficacy and symptom improvement during exposure-based treatment (Bouchard et al., 2007; Gallagher et al., 2013). Given that extinction learning might be analogous to exposure, our findings implicate that differences in self-efficacy levels prior to exposure can mediate anxiety reduction during and after exposure treatment. Moreover, our results challenge the notion that verbal persuasion is less important than mastery experience in increasing perceived self-efficacy (but see Bandura, 1997; Gallagher et al., 2013). In line with the theory of positive and negative cognitions in anxiety (Casey et al., 2004), our findings rather indicate that increasing patients' perceived self-efficacy via social persuasion might constitute an underestimated yet powerful strategy to increase exposure therapy efficacy.

Several limitations of the current study should be considered. First, we did not employ a verbal feedback in the “control condition.” Hence, it cannot be excluded that the verbal feedback *per se* (independent of its positive valence) might have had a similar effect on extinction. The rationale behind this experimental design was that we anticipated a “neutral feedback on self-efficacy” to induce a state of “uncertainty” in our participants. However, since our aim was to test whether extinction can be further enhanced through verbal persuasion and hereby provide a direct implication for exposure treatments, such a control condition might not be equivalent to exposure under standardized conditions (i.e., treatment as usual). A replication of this study with an experimental design, which includes another group of participants, who receive a “neutral feedback” with respect to self-efficacy, would be helpful.

Second, our data suggest that the experimental induction used was not sufficient to elicit differences in self-efficacy between the experimental and control group on the RAS questionnaire (but see Brown et al., 2012a,b). Here, the absence of such an effect might be related to differences in the methodological approach. In contrast to the study by Brown et al. (2012b), we did not use a low self-efficacy induction as a control condition which would probably lead to more pronounced group effects on the RAS measure.

Third, the translation of our findings to useful applications in clinical populations and the therapy setting remains to be further explored. In the present study, we included non-clinical subjects who did not report current or previous mental diseases, psychological or pharmacological treatment for mental diseases, as well as severe acute or chronic somatic diseases. While these criteria were checked prior to the experimentation phase, we did not use a psychodiagnostic interview to assess possible psychiatric diagnoses. Hence, the existence of diagnostically relevant mental health problems in our participants cannot be fully excluded An important extension for future studies would be to examine whether self-efficacy can also be enhanced in patients diagnosed with emotional disorders to counteract deficits in extinction learning (see Blechert et al., 2007; Michael et al., 2007; Briscione et al., 2014). Furthermore, it would be valuable to examine whether the efficacy of exposure-based treatments can be enhanced via modification of self-efficacy beliefs. We suggest that a false positive verbal persuasion would probably be less appropriate to investigate the role of self-efficacy on exposure therapy outcome. However, perceived self-efficacy can be increased via different sources (Bandura, 1997; Maddux, 1999). For instance, one could investigate how positive future imaging or the instructed retrieval of positive self-efficacy experiences (i.e., episodic memories and episodic future thinking, see Zlomuzica et al., 2014) affects the patient's emotional and behavioral responding during exposure treatment.

Finally, we are aware that extinction is an oversimplified model of exposure. While we acknowledge extinction as a major candidate for explaining the effects of exposure, there are several other relevant factors (Margraf and Zlomuzica, 2015) which should not be neglected in potential future clinical studies.

In summary, the present results suggest that fear extinction can be facilitated via positive manipulation of perceived self-efficacy. To our knowledge, we herein show for the first time that perceived self-efficacy alters fear extinction learning and add new evidence on the role of self-efficacy as an important mediator of learning and memory. Our findings might not only provide novel insights into the mechanisms underlying changes in self-efficacy and symptom improvement during exposure, but also trigger new ideas on how cognitive top-down modulation strategies can be used to improve CBT efficacy (Craske et al., 2008, 2014; Vervliet et al., 2013; Margraf and Zlomuzica, 2015).

### Conflict of interest statement

The authors declare that the research was conducted in the absence of any commercial or financial relationships that could be construed as a potential conflict of interest.

## References

[B1] Bandura A. (1997). Self-efficacy: The Exercise of Control. New York, NY: Freeman.

[B2] Berry J. M. (1999). Memory self-efficacy in its social cognitive context, in Social Cognition and Aging, eds Hess T. M. Blanchard-Fields F. (San Diego, CA: Academic Press), 70–96.

[B3] Berry J. M. West R. L. Dennehey D. M. (1989). Reliability and validity of the memory self-efficacy questionnaire. Dev. Psychol. 25, 701–713. 10.1037/0012-1649.25.5.701

[B4] Blechert J. Michael T. Vriends N. Margraf J. Wilhelm F. H. (2007). Fear conditioning in posttraumatic stress disorder: evidence for delayed extinction of autonomic, experiential, and behavioural responses. Behav. Res. Ther. 45, 2019–2033. 10.1016/j.brat.2007.02.012 17442266

[B5] Bouchard S. Gauthier J. Nouwen A. Ivers H. Valliéres A. Simard S.. (2007). Temporal relationship between dysfunctional beliefs, self-efficacy and panic apprehension in the treatment of panic disorder with agoraphobia. J. Behav. Ther. Exp. Psychiatry 38, 275–292. 10.1016/j.jbtep.2006.08.002 17157264

[B6] Bouffard-Bouchard T. (1990). Influence of self-efficacy on performance in a cognitive task. J. Soc. Psychol. 130, 353–363. 10.1080/00224545.1990.9924591

[B7] Briscione M. A. Jovanovic T. Norrholm S. D. (2014). Conditioned fear associated phenotypes as robust, translational indices of trauma-, stressor-, and anxiety-related behaviors. Front. Psychiatry 5:88. 10.3389/fpsyt.2014.00088 25101010 PMC4104832

[B8] Brown A. D. Dorfman M. L. Marmar C. R. Bryant R. A. (2012a). The impact of perceived self-efficacy on mental time travel and social problem solving. Conscious. Cogn. 21, 299–306. 10.1016/j.concog.2011.09.023 22019214

[B9] Brown A. D. Joscelyne A. Dorfman M. L. Marmar C. R. Bryant R. A. (2012b). The impact of perceived self-efficacy on memory for aversive experiences. Memory 20, 374–383. 10.1080/09658211.2012.667110 22424296

[B10] Buhle J. T. Silvers J. A. Wager T. D. Lopez R. Onyemekwu C. Kober H.. (2014). Cognitive reappraisal of emotion: a meta-analysis of human neuroimaging studies. Cereb. Cortex 24, 2981–2990. 10.1093/cercor/bht154 23765157 PMC4193464

[B11] Casey L. M. Oei T. P. S. Newcombe P. A. (2004). An integrated cognitive model of panic disorder: the role of positive and negative cognitions. Clin. Psychol. Rev. 24, 529–555. 10.1016/j.cpr.2004.01.005 15325744

[B12] Comunian A. L. (1989). Some characteristics of relations among depression, anxiety, and self-efficacy. Percept. Mot. Skills 69, 755–764. 10.2466/pms.1989.69.3.755 2608390

[B13] Craske M. G. Kircanski K. Zelikowsky M. Mystkowski J. Chowdhury N. Baker A. (2008). Optimizing inhibitory learning during exposure therapy. Behav. Res. Ther. 46, 5–27. 10.1016/j.brat.2007.10.003 18005936

[B14] Craske M. G. Treanor M. Conway C. C. Zbozinek T. Vervliet B. (2014). Maximizing exposure therapy: an inhibitory learning approach. Behav. Res. Ther. 58, 10–23. 10.1016/j.brat.2014.04.006 24864005 PMC4114726

[B15] Delgado M. R. Nearing K. I. LeDoux J. E. Phelps E. A. (2008). Neural circuitry underlying the regulation of conditioned fear and its relation to extinction. Neuron 59, 829–838. 10.1016/j.neuron.2008.06.029 18786365 PMC3061554

[B16] Delsignore A. Carraro G. Mathier F. Znoj H. Schnyder U. (2008). Perceived responsibility for change as an outcome predictor in cognitive−behavioural group therapy. Br. J. Clin. Psychol. 47, 281–293. 10.1348/014466508X279486 18248693

[B17] Eyring J. D. Johnson D. S. Francis D. J. (1993). A cross-level units-of-analysis approach to individual differences in skill acquisition. J. Appl. Psychol. 78, 805–814. 10.1037/0021-9010.78.5.805 8253633

[B18] Fisk J. E. Warr P. (1996). Age-related impairment in associative learning: the role of anxiety, arousal and learning self-efficacy. Pers. Individ. Dif. 21, 675–686. 10.1016/0191-8869(96)00120-1

[B19] Gallagher M. W. Payne L. A. White K. S. Shear K. M. Woods S. W. Gorman J. M.. (2013). Mechanisms of change in cognitive behavioral therapy for panic disorder: the unique effects of self-efficacy and anxiety sensitivity. Behav. Res. Ther. 51, 767–777. 10.1016/j.brat.2013.09.001 24095901 PMC3866809

[B20] Gaudiano B. A. Herbert J. D. (2007). Self-efficacy for social situations in adolescents with generalized social anxiety disorder. Behav. Cogn. Psychother. 35, 209–223. 10.1017/S1352465806003377

[B21] Goldin P. R. Ziv M. Jazaieri H. Werner K. Kraemer H. Heimberg R. G.. (2012). Cognitive reappraisal self-efficacy mediates the effects of individual cognitive-behavioral therapy for social anxiety disorder. J. Consult. Clin. Psychol. 80, 1034–1040. 10.1037/a0028555 22582765 PMC3424305

[B22] Gwaltney C. J. Shiffman S. Balabanis M. H. Paty J. A. (2005). Dynamic self-efficacy and outcome expectancies: prediction of smoking lapse and relapse. J. Abnorm. Psychol. 114, 661–675. 10.1037/0021-843X.114.4.661 16351387

[B23] Haselgrove M. Aydin A. Pearce J. M. (2004). A partial reinforcement extinction effect despite equal rates of reinforcement during pavlovian conditioning. J. Exp. Psychol. Anim. Behav. Process 30, 240–250. 10.1037/0097-7403.30.3.240 15279514

[B24] Hertzog C. Dixon R. A. Hultsch D. F. (1990). Relationships between metamemory, memory predictions, and memory task-performance in adults. Psychol. Aging 5, 215–227. 10.1037/0882-7974.5.2.215 2378687

[B25] Johnson J. Gooding P. A. Wood A. M. Tarrier N. (2010). Resilience as positive coping appraisals: testing the schematic appraisals model of suicide (SAMS). Behav. Res. Ther. 48, 179–186. 10.1016/j.brat.2009.10.007 19906364

[B26] Kalpouzos G. Eriksson J. (2013). Memory self-efficacy beliefs modulate brain activity when encoding real-world future intentions. PLoS ONE 8:e73850. 10.1371/journal.pone.0073850 24019938 PMC3760799

[B27] Lent R. W. Brown S. D. Gore P. A. (1997). Discriminant and predictive validity of academic self-concept, academic self-efficacy, and mathematics-specific self-efficacy. J. Couns. Psychol. 44, 307–315. 10.1037/0022-0167.44.3.307

[B28] Lent R. W. Brown S. D. Larkin K. C. (1986). Self-efficacy in the prediction of academic performance and perceived career options. J. Couns. Psychol. 33, 265 10.1037/0022-0167.33.3.265

[B29] Lovibond S. H. Lovibond P. F. (1995). Manual for the Depression Anxiety Stress Scales. Sydney, NSW: Psychology Foundation.

[B30] Maddux J. E. (1999). Expectancies and the social-cognitive perspective: basic principles, processes, and variables, in How Expectancies Shape Behavior, ed Kirsch I. (Washington, DC: American Psychological Association), 17–40.

[B31] Margraf J. Zlomuzica A. (2015). Changing the future, not the past: a translational paradigm shift in treating anxiety. EMBO Rep. 16, 259–260. 10.15252/embr.201540076 25662154 PMC4364861

[B32] Marlatt G. A. Donovan D. M. (2005). Relapse Prevention: Maintenance Strategies in the Treatment of Addictive Behaviors. New York, NY: GuilfordPress.

[B33] Marquez D. X. Jerome G. J. McAuley E. Snook E. M. Canaklisova S. (2002). Self-efficacy manipulation and state anxiety responses to exercise in low active women. Psychol. Health 17, 783–791. 10.1080/0887044021000054782

[B34] McDougall G. J. (1998). Increasing memory self-efficacy and strategy use in Hispanic elders. Clin. Gerontol. 19, 57–76. 10.1300/J018v19n02_05 18802497 PMC2542897

[B35] McDougall G. J. Kang J. (2003). Memory self-efficacy and memory performance in older males. Int. J. Mens. Health 2, 131–147. 10.3149/jmh.0202.131 19043600 PMC2586289

[B36] McFarlane A. H. Bellissimo A. Norman G. R. (1995). The role of family and peers in social self-efficacy - links to depression in adolescence. Am. J. Orthopsychiat. 65, 402–410. 10.1037/h0079655 7485425

[B37] Michael T. Blechert J. Vriends N. Margraf J. Wilhelm F. H. (2007). Fear conditioning in panic disorder: enhanced resistance to extinction. J. Abnorm. Psychol. 116, 612–617. 10.1037/0021-843X.116.3.612 17696717

[B38] Mitchell T. R. Hopper H. Daniels D. Georgefalvy J. James L. R. (1994). Predicting self-efficacy and performance during skill acquisition. J. Appl. Psychol. 79, 506–517. 10.1037/0021-9010.79.4.506

[B39] Moritz S. E. Feltz D. L. Fahrbach K. R. Mack D. E. (2000). The relation of self-efficacy measures to sport performance: a meta-analytic review. Res. Q. Exerc. Sport 71, 280–294. 10.1080/02701367.2000.10608908 10999265

[B40] Mosig C. Merz C. J. Mohr C. Adolph D. Wolf O. T. Schneider S.. (2014). Enhanced discriminative fear learning of phobia-irrelevant stimuli in spider-fearful individuals. Front. Behav. Neurosci. 8:328. 10.3389/fnbeh.2014.00328 25324745 PMC4181334

[B41] Norrholm S. D. Jovanovic T. (2010). Tailoring therapeutic strategies for treating posttraumatic stress disorder symptom clusters. Neuropsychiatr. Dis. Treat. 6, 517–532. 10.2147/NDT.S10951 20856915 PMC2938301

[B42] Paunonen S. V. Hong R. Y. (2010). Self-efficacy and the prediction of domain-specific cognitive abilities. J. Pers. 78, 339–359. 10.1111/j.1467-6494.2009.00618.x 20433622

[B43] Payne B. R. Jackson J. J. Hill P. L. Gao X. F. Roberts B. W. Stine-Morrow E. A. L. (2012). Memory self-efficacy predicts responsiveness to inductive reasoning training in older adults. J. Gerontol. B Psychol. 67, 27–35. 10.1093/geronb/gbr073 21743037 PMC3267022

[B44] Richards J. C. Richardson V. Pier C. (2002). The relative contributions of negative cognitions and self-efficacy to severity of panic attacks in panic disorder. Behav. Change 19, 102–111. 10.1375/bech.19.2.102

[B45] Rothbaum B. O. Davis M. (2003). Applying learning principles to the treatment of post-trauma reactions. Ann. N.Y. Acad. Sci. 1008, 112–121. 10.1196/annals.1301.012 14998877

[B46] Ruhmland M. Margraf J. (2001a). Effektivität psychologischer therapien von generalisierter angststörung und sozialer phobie: meta-analysen auf störungsebene. Verhaltenstherapie 11, 27–40. 10.1159/000050322

[B47] Ruhmland M. Margraf J. (2001b). Effektivität psychologischer therapien von panik und agoraphobie: meta-analysen auf störungsebene. Verhaltenstherapie 11, 41–53. 10.1159/000050323

[B48] Ruhmland M. Margraf J. (2001c). Effektivität psychologischer therapien von spezifischer phobie und zwangsstörung: meta-analysen auf störungsebene. Verhaltenstherapie 11, 14–26. 10.1159/000050321

[B49] Schiller D. Delgado M. R. (2010). Overlapping neural systems mediating extinction, reversal and regulation of fear. Trends Cogn. Sci. 14, 268–276. 10.1016/j.tics.2010.04.002 20493762 PMC3848321

[B50] Schnoll R. A. Martinez E. Tatum K. L. Glass M. Bernath A. Ferris D.. (2011). Increased self-efficacy to quit and perceived control over withdrawal symptoms predict smoking cessation following nicotine dependence treatment. Addict. Behav. 36, 144–147. 10.1016/j.addbeh.2010.08.024 20869812 PMC2981675

[B51] Schwarzer R. Fuchs R. (1995). Changing risk behaviors and adopting health behaviors: the role of self-efficacy beliefs, in Self-efficacy in Changing Societies, ed Bandura A. (New York, NY: Cambridge University Press), 259–288. 10.1017/CBO9780511527692.011

[B52] Seeman T. McAvay G. Merrill S. Albert M. Rodin J. (1996). Self-efficacy beliefs and change in cognitive performance: MacArthur studies on successful aging. Psychol. Aging 11, 538–551. 10.1037/0882-7974.11.3.538 8893321

[B53] Stajkovic A. D. Luthans F. (1998). Self-efficacy and work-related performance: a meta-analysis. Psychol. Bull. 124, 240 10.1037/0033-2909.124.2.240

[B54] Thomasson P. Psouni E. (2010). Social anxiety and related social impairment are linked to self-efficacy and dysfunctional coping. Scand. J. Psychol. 51, 171–178. 10.1111/j.1467-9450.2009.00731.x 19500297

[B55] Vervliet B. Craske M. G. Hermans D. (2013). Fear extinction and relapse: state of the art. Annu. Rev. Clin. Psychol. 9, 215–248. 10.1146/annurev-clinpsy-050212-185542 23537484

[B56] Vögele C. Ehlers A. Meyer A. H. Frank M. Hahlweg K. Margraf J. (2010). Cognitive mediation of clinical improvement after intensive exposure therapy of agoraphobia and social phobia. Depress. Anxiety 27, 294–301. 10.1002/da.20651 20037922

[B57] Vriends N. Michael T. Blechert J. Meyer A. H. Margraf J. Wilhelm F. H. (2011). The influence of state anxiety on the acquisition and extinction of fear. J. Behav. Ther. Exp. Psy. 42, 46–53. 10.1016/j.jbtep.2010.09.001 21074006

[B58] Williams S. L. (1995). Self-efficacy, anxiety, and phobic disorders, in Self-efficacy, Adaptation, and Adjustment: Theory, Research and Application, ed Maddux J. E. (New York, NY: Plenum Press), 69–107.

[B59] Zlomuzica A. Dere D. Machulska A. Adolph D. Dere E. Margraf J. (2014). Episodic memories in anxiety disorders: clinical implications. Front. Behav. Neurosci. 8:131. 10.3389/fnbeh.2014.00131 24795583 PMC4005957

